# Through-Wall UWB Radar Based on Sparse Deconvolution with Arctangent Regularization for Locating Human Subjects

**DOI:** 10.3390/s21072488

**Published:** 2021-04-03

**Authors:** Artit Rittiplang, Pattarapong Phasukkit

**Affiliations:** School of Engineering, King Mongkut’s Institute of Technology Ladkrabang, Bangkok 10520, Thailand; 59601306@kmitl.ac.th

**Keywords:** sparse deconvolution, majorization–minimization (MM) algorithm, arctangent regularization, through-wall radar, UWB radar

## Abstract

A common problem in through-wall radar is reflected signals much attenuated by wall and environmental noise. The reflected signal is a convolution product of a wavelet and an unknown object time series. This paper aims to extract the object time series from a noisy receiving signal of through-wall ultrawideband (UWB) radar by sparse deconvolution based on arctangent regularization. Arctangent regularization is one of the suitably nonconvex regularizations that can provide a reliable solution and more accuracy, compared with convex regularizations. An iterative technique for this deconvolution problem is derived by the majorization–minimization (MM) approach so that the problem can be solved efficiently. In the various experiments, sparse deconvolution with the arctangent regularization can identify human positions from the noisy received signals of through- wall UWB radar. Although the proposed method is an odd concept, the interest of this paper is in applying sparse deconvolution, based on arctangent regularization with an S-band UWB radar, to provide a more accurate detection of a human position behind a concrete wall.

## 1. Introduction

Basic deconvolution is the process of extracting the unknown input signal (**x**) of a linear time-invariant system (**y** = **Hx** in matrix form) when the noise-free output signal (**y**) and wavelet (**H**) are known. However, in real-world applications, the output signal (**y**) is noisy and distorted by inhomogeneous media, such as ground-penetrating radar (GPR) [1,2], seismicity [3,4], radars [5,6,7,8], astronomy [9], speech recognition [10,11], and image reconstruction [12,13,14,15]. Nowadays, sparse deconvolution plays an important role in extracting the original data from the noisy received signal; it has been widely used in denoising, interpolation, super-resolution, and declipping [16,17,18,19,20,21,22,23,24,25,26]. Whereas linear time-invariant (LTI) filters, such as low-pass, band-pass and high pass, have amplitude distortions on the original signal resolution [27,28,29,30,31,32,33,34,35].

The sparse deconvolution algorithm is a numerical method to restore the original signal by optimization formulation with L1 norm regularization. L1 norm regularization could effectively remove noise and rectify parts of signal distortion [21,22,23,24,25,26,27,28,29,30,31,32,33,34,35,36]. Meanwhile, L2 norm regularization fails to remove noise, while aggravating signal distortion, and the Lp pseudo-norm regularization (0 < *p* < 1) completely removes noise and corrects most of the signal distortion [20,21]. However, the Lp pseudo-norm suffers from nonconvex optimization, which, in turn, leads to the erroneous local optimal point of the cost function [37].

As a result, this research utilizes a nonconvex arctangent regularization function which is parameterized by a parameter tuner to avoid the nonconvex optimization of the cost function, and, thus, realize the local optimal point [32,33,34,35,36,37]. This advantage will be applied in through-wall UWB radar application, in this paper, to extract human ranges behind a wall from a noisy received signal. The advantages of UWB radars, which work by emitting short pulses of high-frequency electromagnetic wave (EM), are that they can provide high penetration, high-range resolution, less harm to human health, and lower power consumption, compared with continuous-wave radars [16]. UWB radars are thus suitable for through-wall applications and the detection of human subjects behind solid objects [38,39,40,41,42,43,44,45,46]. The S-band frequency range (2–4 GHz) was used in the proposed radar scheme to provide both high spatial resolution and wall penetration [38,39,40,41,42,43,44,45,46,47,48].

In previous works [43,44,48], UWB radars identified human objects behind a wall by detecting respiration rate. However, UWB radar algorithms for respiration and/or heartbeat detection need to capture received signals at least 512 times per minute (60 s), for over 1 cycle vital sign signal, with Nyquist sampling condition. As a result, we are interested in the sparse deconvolution algorithm with arctangent regularization for reconstruing human range from only one received signal, and for faster detection. This method provides high performance for detecting humans by range movement, but it is difficult to distinguish between a standing human and static objects. In real-world applications, humans have motions so that the detectable range is sufficient for obtaining their positions [41].

The organization of this research is as follows: Section 1 is the introduction. Section 2 details the theoretical background of convolution, deconvolution, and sparse deconvolution. Section 3 describes sparse deconvolution with arctangent regularization and the majorization–minimization algorithm. Section 4 deals with the experimental setup and results in the detection of human subjects behind a wall. The concluding remarks are provided in Section 5.

## 2. Theoretical Background

We begin this paper with necessary background knowledge for reconstructing target signals in through-the-wall UWB radar application, which briefly introduces convolution, deconvolution, and sparse deconvolution with convex and nonconvex functions with the MM algorithm.

### 2.1. Convolution Model

**Figure 1:**
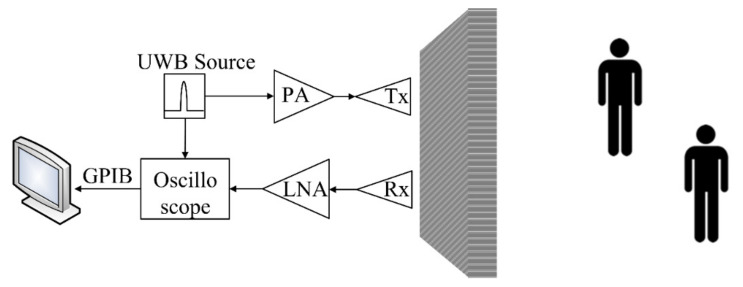
Block diagram of the through-wall UWB radar scheme.

A block diagram of the UWB radar system is experimentally determined in Figure 1. When the transmitter emits, part of the energy is reflected off the wall, and objects detected by receivers are then captured by an oscilloscope [43,44,45].

A recorded radar data is a linear system where a UWB wavelet *h*(*t*) is convolved with the reflectivity series *x*(*t*). In practice, the radar data (**y**) can be expressed in a matrix form with environmental noise (see details in [1]).

(1) y=Hx+w

For complexity reduction of matrix inversion, the reflectivity series (**x**) will be assumed to be the same size of **y**, where $y\in {\mathbb{R}}^{N}$ is the received signal in vector form,$\;x\in {\mathbb{R}}^{N}$ is the reflectivity series (sparse signal) in vector form, $w\in {\mathbb{R}}^{N}$ is white Gaussian noise in matrix form, and $H\in \;\mathbb{R}{\;}^{N\times N}$ is a convolution matrix.

(2) $$H=\left[\begin{array}{ccccc} h_{0} & 0 & 0 & \cdots & 0\\ h_{1} & h_{0} & 0 & \ddots & 0\\ h_{0} & h_{1} & \ddots & \ddots & \vdots \\ \vdots & \ddots & \ddots & h_{0} & 0\\ h_{N-1} & \cdots & h_{2} & h_{1} & h_{0} \end{array}\right]=A^{-1}B$$

The convolution matrix **H** is a Toeplitz structure and determined as **H** = **A**^−1^**B**, where **A** and **B** are band matrices (sparse matrix) ∈ ℝ^N×N^ [36]. If the matrices **A** and **B** are so far from the band matrix, such as N = 2, then also $A^{-1}B$**,** based on the Equation (2), is so far from the exact result.

(3) $$A=\left[\begin{array}{cccccc} a_{0} & 0 & 0 & \vdots & 0 & 0\\ a_{1} & a_{0} & 0 & \ddots & \ddots & 0\\ \vdots & a_{1} & \ddots & \ddots & \ddots & \vdots \\ a_{j} & \vdots & \ddots & a_{0} & 0 & 0\\ 0 & \ddots & \vdots & a_{0}a_{1}0\\ 0 & 0 & a_{j} & \cdots & a_{1} & a_{0} \end{array}\right]$$

(4) $$B=\left[\begin{array}{cccccc} b_{0} & 0 & 0 & \vdots & 0 & 0\\ b_{1} & b_{0} & 0 & \ddots & \ddots & 0\\ \vdots & b_{1} & \ddots & \ddots & \ddots & \vdots \\ b_{i} & \vdots & \ddots & b_{0} & 0 & 0\\ 0 & \ddots & \ddots & b_{0}b_{1}0\\ 0 & 0 & b_{i} & \cdots & b_{1} & b_{0} \end{array}\right]$$

where the matrix elements of **A** and **B** consist of *a_j_* and *b_i_* coefficients. The *a_j_* and *b_i_* are derived from the *Z*-transform of the wavelet (Gaussian pulse) *h*(n), where $h\left(n\right)=nr^{n}\sin\left(\omega_{0}n\right)$ for analysis of **A** and **B** band matrices [36]. The *Z*-transform of the wavelet is mathematically expressed.

(5) $$Z\left\{h\left(n\right)\right\}=Z\left\{nr^{n}\sin\left(\omega_{0}n\right)\right\}=Z\left\{nf\left(n\right)\right\}=-z\frac{dF\left(z\right)}{dz}$$

where $f\left(n\right)=r^{n}\sin\left(\omega_{0}n\right)↔F\left(z\right)=r\sin\left(\omega_{0}n\right)Z^{-1}/\left(1-2r\cos\left(\omega_{0}n\right)Z^{-1}+r^{2}Z^{-2}\right)$ given 0 < |r| < 1. Substituting *F*(*z*) to $-zdF\left(z\right)/dz$ in the Equation (5), we have

(6) $$Z\left\{nr^{n}\sin\left(\omega_{0}n\right)\right\}=\frac{r\sin\left(\omega_{0}\right)Z^{-1}-r^{3}\sin\left(\omega_{0}\right)Z^{-3}}{1-4r\cos\left(\omega_{0}\right)Z^{-1}+\left\{4r^{2}\cos^{2}\left(\omega_{0}\right)+2r^{2}\right\}Z^{-2}-4r^{3}\cos\left(\omega_{0}\right)Z^{-3}+r^{4}Z^{-4}}=\frac{b}{a}$$

The Equation (6) is of four order $\left(Z^{-4}\right)$ and the coefficients *a_j_* and *b_i_* are derived from the denominator and numerator in Equation (6)

(7) $$a=\left[a_{0},a_{1},a_{2},a_{3},a_{4}\right]=\left[1,-4r\cos\left(\omega_{0}\right),4r^{2}\cos^{2}\left(\omega_{0}\right)+2r^{2},-4r^{3}\cos\left(\omega_{0}\right),r^{4}\right]$$

and

(8) $$b=\left[b_{0},b_{1},b_{2},b_{3}\right]=\left[0,r\sin\left(\omega_{0}\right),0,-r^{3}\sin\left(\omega_{0}\right)\;\right]$$

where *r* is the pole radius and *ω*_0_ is a normalized angular frequency (*ω*_0_ = *ω/fs*) as a function of the transmitting frequency (*ω =* 2πf) and the sampling frequency (*fs*).

**Figure 2:**
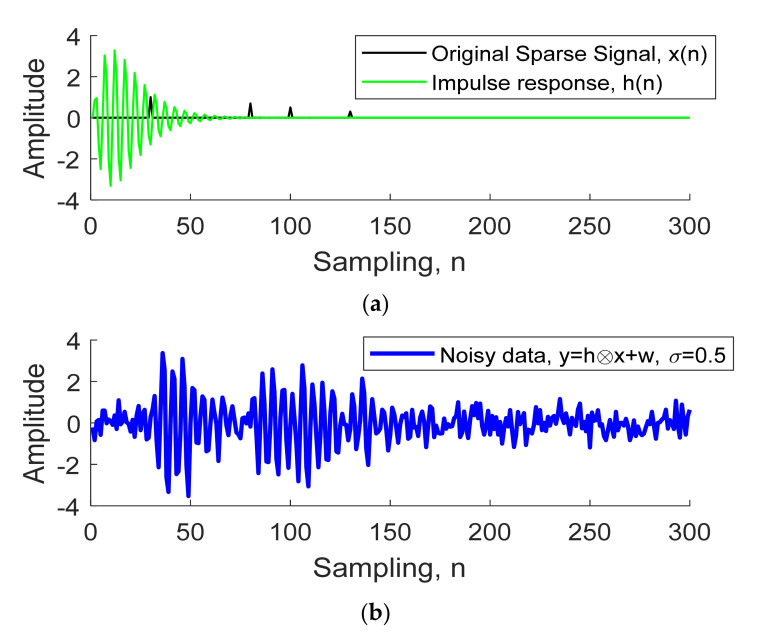
Reflected received signal of through-wall UWB radar, given three behind-the-wall objects: (**a**) the reflectivity series and wavelet (impulse response), (**b**) noisy signal.

Figure 2a illustrates the reflectivity series x(n) (black line) and wavelet h(n) (green line) of UWB radar with three behind-the-wall objects. Given the reflectivity series x(n) = δ (n − 30) + 0.7δ (n − 80) + 0.5δ (n − 100) + 0.3δ (n − 130) with other indexes x(n) = 0, the first spike is assumed as the wall reflection. Figure 2b shows the noisy signal (y=Hx+w). The coefficients *a_j_* and *b_i_* were derived from Equations (7) and (8) to create the convolution matrix **H**, given *r* = 0.9 and *ω*_0_ = 0.4π. The standard deviation (σ) of white Gaussian noise (**w**) was 0.5. We aim to find the object time series from the noisy data **y**, described in the next section.

### 2.2. Deconvolution Model

Deconvolution is estimating **x** from **y** (**y** = **Hx** + **w**), but **x** cannot be directly solved by rearranging in the form of **x** = **H**^−1^**(y**−**w)** or **x** ≈ **H^−^**^1^**y**, because **H** may be singular and non-square matrices that **H** has no inverse form [1,2,3,4,5,6,7,8,9,10,11,12,13,14,15,16,17,18,19,20,21,22]. The alternative approach to estimating **x** is by minimizing the cost function $F\left(x\right)=||y-H{x||}_{2}^{2}$. The estimated signal $\hat{x}$ is derived by optimizing the cost function.

(9) $$\hat{x}=\underset{x\in {\mathbb{R}}^{N}}{\mathrm{argmin}}||y-H{x||}_{2}^{2}={\left(H^{T}H\right)}^{-1}H^{T}y\;$$

The estimated signal $\hat{x}$ is as consistent with **y** as possible, according to the square error criterion; however, **H**^T^**H** may not be invertible. To avoid this problem, we must add a regularization term into the cost function; as shown below, it is the solution to the more general problem [20,41].

(10) $$F\left(x\right)=\underset{\mathrm{closed}\;\mathrm{convex}\;\mathrm{function}}{\underset{︸}{||y-H{x||}_{2}^{2}}}+\underset{\mathrm{regularization}}{\underset{︸}{\lambda \underset{n}{\sum }\phi \left(x\left(n\right)\right)}}$$

It is the cost function in the general form to solve the solution **x**, as well known $x=\;\mathrm{argmin}\;F\left(x\right)$, where $||y-H{x||}_{2}^{2}$ is a closed convex function to force **x** to be consistent with the measured signal **y**. 𝜙(x) is a regularization term (or penalty function) that can enhance some desired features of **x**, and *λ* is the regularization parameter that balances the importance of data fidelity about the estimated signal **x**.

In the case of the low noise received signal **y**, we can roughly estimate the reflectivity series **x** by the optimization problem, Equation (10), with the regularization term of small energy (L2 norm) [1,2,3,4,5,6,7,8,9,10,11,12,13,14,15,16,17,18,19,20,21,22], $\underset{n}{\sum }\phi \left(x\left(n\right)\right)={\left||x|\right|}_{2}^{2}$.

(11) $$\hat{x}=\underset{x\in {\mathbb{R}}^{N}}{\mathrm{argmin}}\left\{||y-H{x||}_{2}^{2}+\underset{L2\;norm}{\underset{︸}{\lambda {\left||x|\right|}_{2}^{2}}}\right\}={\left(H^{T}H+\lambda I\right)}^{-1}H^{T}y$$

where **I** is the identity matrix. The notation $x_{2}^{2}$ represents the sum of squares of the vector **x**, $x_{2}^{2}={\left|x_{1}\right|}^{2}+{\left|x_{2}\right|}^{2}+\ldots +{\left|x_{N}\right|}^{2}$.

### 2.3. Sparse Deconvolution Model with L1 Norm (Convex Regularization)

Given the limitation of L2 norm regularization and the noisy characteristic of received signal **y**, we can easily estimate **x** to be a sparse signal (spike) from **y** by minimizing Equation (10) with a convex regularization term of the L1 norm [1,2,3,4,5,6,7,8,9,10,11,12,13,14,15,16,17,18,19,20,21,22], $\underset{n}{\sum }\phi \left(x\left(n\right)\right)=x_{1}$.

(12) $$\hat{x}=\underset{x\in {\mathbb{R}}^{N}}{\mathrm{argmin}}\left\{||y-H{x||}_{2}^{2}+\underset{L1\;norm}{\underset{︸}{\lambda ||{x||}_{1}}}\right\}$$

where $x_{1}$ is called L1 norm regularization (convex regularization) represented by the sum of absolute values of vector **x**, $x_{1}=\left|x_{1}\right|+\left|x_{2}\right|+\left|x_{3}\right|+\ldots +\left|x_{N}\right|$. The Equation (12) is the sparse signal deconvolution problem in a simple form; this problem cannot be solved in an explicit form, because the cost function is not differentiable. It must be solved by using an iterative numerical algorithm; this research used the majorization–minimization (MM) algorithm, which will be discussed in the next section.

## 3. Sparse Deconvolution Method

This section has been divided into three parts. Section 3.1 and Section 3.2 propose the arctangent regularization and numerical method for this sparse deconvolution, respectively. The last part presents a flowchart of the proposed method for through-wall UWB radar.

### 3.1. Sparse Deconvolution with Arctangent Regularization (Non-Convex Regularization)

To improve signal sparsity, the L1 norm (convex) regularization in Equation (12) could be replaced with a nonconvex regularization [20,21,22]. In this paper, the nonconvex arctangent regularization in Equation (13) was used in place of the L1 norm to improve the detection performance of the through-wall UWB radar scheme. Arctangent regularization can provide reliable minimization and a fast solution using the MM algorithm, see Section II-E in [37]. The arctangent function $\phi_{\mathrm{atan}}\left(x,\alpha \right)$ is a parameterized regularization with *α >* 0 (e.g., logarithmic and first-order ration) and is mathematically expressed as

(13) $$\phi_{\mathrm{atan}}\left(x,\alpha \right)=\left\{\begin{array}{l} \frac{2}{\alpha \sqrt{3}}\left(\tan^{-1}\left(\frac{1+2\alpha \left|x\right|}{\sqrt{3}}\right)-\frac{\pi }{6}\right),\;\alpha >0\\ \left|x\right|\;\;\;\;\;\;\;\;\;\;\;\;\;\;\;\;\;\;\;\;\;\;\;\;\;\;\;\;\;\;\;\;\;\;\;\;\;\;\;\;,\;\alpha \;\approx 0 \end{array}\right.$$

The derivative of $\phi_{\mathrm{atan}}$ is written as shown below, to prepare in the MM algorithm.

(14) $$\phi_{\mathrm{atan}}^{\prime }\left(x,\alpha \right)=\left\{\begin{array}{l} \frac{1}{\alpha^{2}x^{2}+\alpha \left|x\right|+1}sign\left(x\right),\;\alpha >0\\ sign\left(x\right)\;\;\;\;\;\;\;\;\;\;\;\;\;\;\;\;\;\;\;,\;\alpha \;\approx 0 \end{array}\right.$$

where 0 < *α* < 1/*λ* is a tuner parameter to avoid nonconvex optimization. The arctangent function is continuous, symmetric, twice differentiable on ℝ\{0}, increasing on ℝ_+_, and concave on ℝ_+_. The right-sided second derivative is $\phi_{\mathrm{atan}}^{\prime \prime }\left(0^{+}\right)=-\alpha$. A sparse deconvolution with arctangent regularization is given by

(15) $$\hat{x}=\underset{x\in {\mathbb{R}}^{N}}{\mathrm{argmin}}\left\{||y-H{x||}_{2}^{2}+\lambda \underset{n}{\sum }\phi_{\mathrm{atan}}\left(x\left(n\right)\right)\right\}$$

### 3.2. Minimizing the Cost Function Using Majorization-Minimization (MM) Approach

**Figure 3:**
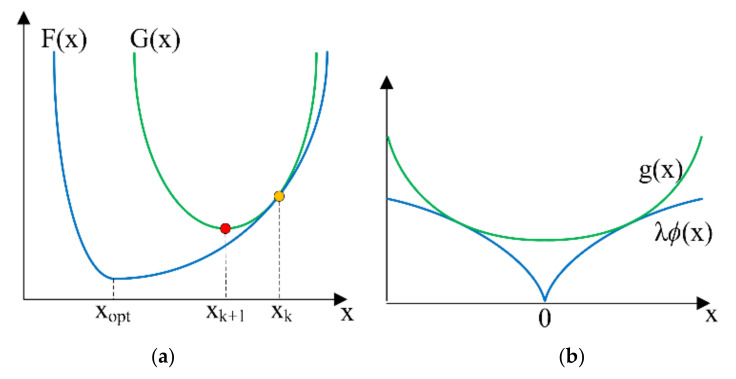
(**a**) Cost function F(x) and its quadratic majorizer G(x); (**b**) regularization function λ𝜙(x) and its quadratic majorizer g(x).

Sparse deconvolution with L1 norm, Equation (12), and arctangent regularization, Equation (15), cannot be carried out directly because both equations are not differentiable. To estimate the sparse signal $\hat{x}$, MM algorithm was utilized to minimize the cost function *F*(*x*) by sequentially minimizing the quadratic majorizer *G*(*x*), as shown in Figure 3a.

This idea is that each *G*(*x*) is easier to solve than *F*(*x*). The MM approach produces a sequence, $x_{k+1}$, each being obtained by minimizing *G*(*x*) and converging to the minimizer of *F*(*x*), where *k* is the iteration counter, *k* = 0, 1, 2, …, *K*. The updated point **x**_k+1_ is derived by minimizing the quadratic majorizer *G*(*x*)

(16) $$x_{k+1}=\underset{x\in {\mathbb{R}}^{N}}{\mathrm{argmin}}G\left(x\right)$$

The function *G*(**x**) must always be the majorizer (upper bound) of the cost function *F*(**x**), $G\left(x\right)\geq F\left(x\right),\;\forall x\in {\mathbb{R}}^{N}$. The tangent point between *G*(**x**_k_) and *F*(**x**_k_) is related by

(17) $$G\left(x_{k}\right)=F\left(x_{k}\right)\;\;\mathrm{and}\;\;G^{\prime }\left(x_{k}\right)=F^{\prime }\left(x_{k}\right)\;$$

In practice, the chosen majorizer *G*(**x**) should be relatively easy to minimize. The easy-to-minimize function *G*(**x**) is written as

(18) $$G\left(x\right)=||y-H{x||}_{2}^{2}+g\left(x\right)=||y-H{x||}_{2}^{2}+x^{T}Q_{k}x+c\left(x_{k}\right)$$

where g(**x**) must be an upper bound for the regularization term λ𝜙(x) in the cost function *F*(**x**), as shown in Figure 3b. $g\left(x\right)=x^{T}Q_{k}x+c\left(x_{k}\right)$, where *c*(**x***_k_*) is constant vectors independent of **x**; and **Q***_k_* is a diagonal matrix,

(19) $$Q_{k}=\lambda *\mathrm{diag}\left(\phi^{\prime }\left(x_{k}\right)./x_{k}\right)\;\;$$

where “diag(.)” is the diagonal matrix operator and the notation ‘./’ denotes component-wise division. Therefore, the MM update in Equation (16) by minimizing Equation (18) with respect to **x** gives

(20) $$x_{k+1}={\left(H^{T}H+Q_{k}\right)}^{-1}H^{T}y$$

Substituting **H** with **A**^−1^**B** obtains

(21) $$x_{k+1}={\underset{\mathrm{not}\;\mathrm{band}\;\mathrm{matrix}}{\underset{︸}{\left(B^{T}{\left(AA^{T}\right)}^{-1}B+Q_{k}\right)}}}^{-1}B^{T}A^{-T}y$$

However, there are two problems, as follows [36]:

(1) The update $x_{k+1}$ (Equation (21)) is mathematically valid, but it may become numerically inaccurate because the entries of **Q*_k_*** go to infinity when the components of $x_{k}$ go to sparse (go to zero).
(2) Its inverse matrix is not banded due to (**AA**^T^)^−1^, which has a high computational cost; so fast solvers cannot be used here.

To address both issues, the matrix inverse lemma was used to alter the non-banded matrix to the banded matrix, given by

(22) $${\underset{\mathrm{not}\;\mathrm{band}\;\mathrm{matrix}}{\underset{︸}{\left(B^{T}{\left(AA^{T}\right)}^{-1}B+Q_{k}\right)}}}^{-1}=Q_{k}^{-1}-Q_{k}^{-1}B^{T}{\underset{\mathrm{band}\;\mathrm{matrix}}{\underset{︸}{\left(AA^{T}+BQ_{k}^{-1}B^{T}\right)}}}^{-1}BQ_{k}^{-1}$$

Substituting Equation (22) into Equation (21) obtains

(23) $$x_{k+1}=Q_{k}^{-1}\left[B^{T}A^{-T}y-B^{T}{\left(AA^{T}+BQ_{k}^{-1}B^{T}\right)}^{-1}BQ_{k}^{-1}B^{T}A^{-T}y\right]$$

It shows that the diagonal matrix $Q_{k}^{-1}$ cannot be infinity, even though $x_{k}$ is approaching the sparse signal solution. The iteration counter $x_{k+1}$ depends on $Q_{k}^{-1}$ starts with the initial value **x**_0_ = **y**.

(24) $$Q_{k}^{-1}=\Lambda =\frac{1}{\lambda }\mathrm{diag}\left(\frac{x_{k}}{\phi^{\prime }\left(x_{k}\right)}\right)=\frac{1}{\lambda }\left[\begin{array}{ccccccc} \frac{x_{k}\left(1\right)}{\phi^{\prime }\left(x_{k}\left(1\right)\right)} & & & & & 0 & \\ & \frac{x_{k}\left(2\right)}{\phi^{\prime }\left(x_{k}\left(2\right)\right)} & & & & & \\ & & \ddots & & & \\ & & & & \ddots & & \\ & & 0 & & & \ddots & \\ & & & & & & \frac{x_{k}\left(N\right)}{\phi^{\prime }\left(x_{k}\left(N\right)\right)} \end{array}\right]$$

where $x_{k}./\phi^{\prime }\left(x_{k}\right)$ for each scalar value of its matrix for L1 norm is given by

(25) $$\frac{x}{\phi^{\prime }\left(x\right)}=\left|x\right|$$

and $x_{k}./\phi^{\prime }\left(x_{k}\right)$ for arctangent function, according to Equation (14), is given by

(26) $$\frac{x}{\phi^{\prime }\left(x,\alpha \right)}=\left|x\right|\left(1+\alpha \left|x\right|+\alpha^{2}{\left|x\right|}^{2}\right)$$

to avoid non-convex optimization, the parameter tuner α is given by

(27) $$0<\alpha <\frac{1}{\lambda }\mathrm{and}\;\lambda \approx 3\mathrm{std}\left(H^{T}w\right)$$

where ‘std’ is the standard deviation of $H^{T}w$, and **w** is the white Gaussian noise [37]. Furthermore, reducing λ leads to an even noisier solution. Increasing λ leads to further attenuation of both the solution and noise. An implementation of the MM algorithm is given in Algorithm 1 to extract a sparse signal from a noisy received signal, where the elements of the diagonal matrix, $x_{k}./\phi^{\prime }\left(x_{k}\right)$, are denoted in Equation (26).
**Algorithm 1.** Sparse Deconvolution with MM Method, Where **A** and **B** Are Formed by Equations (3) and (4).
Input: **y** ∈ ℝ^N^, **A**, and **B** ∈ ℝ^N×N^, λ, $x/\phi^{\prime }\left(x\right)$ Output **x** ∈ ℝ^N^ **x** = **y** (initialization)
$g=B^{T}A^{-T}y$ repeat $\Lambda \leftarrow \frac{1}{\lambda }\mathrm{diag}\left(\frac{x\left(n\right)}{\phi^{\prime }\left(x\left(n\right)\right)}\right)$ (diag (.) is diagonal matrix operator and *x*(n) is N samples of **x**) $F=AA^{T}+B\Lambda B^{T}$ $x=\;\Lambda g-\Lambda B^{T}F^{-1}B\Lambda g$ until convergence (optimization problem Equation (10) has F(x_k+1_) − F(x_k_) < 0.01) return **x**

**Figure 4:**
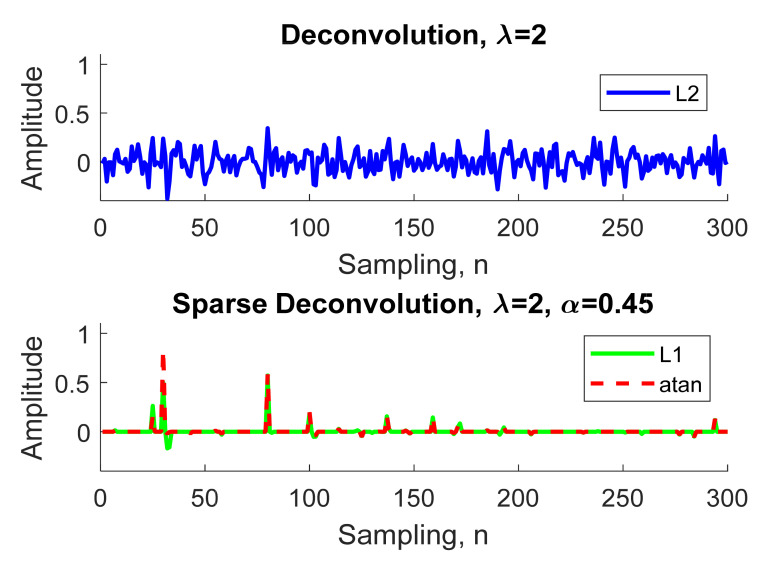
Comparison between the estimated sparse signal using deconvolution with L2 norm, L1 norm, and arctangent regularizations.

We illustrate an example in Figure 4 that, to extract the object time series from the noisy signal in Figure 2, the reflectivity series **x** was assumed to have four objects, **x** = δ (n − 30) + 0.7δ (n − 80) + 0.5δ (n − 100) + 0.3δ (n − 130). Given λ = 2, we compare the normal deconvolution by L2 norm Equation (11) to both sparse deconvolutions by the L1 norm Equation (12) and arctangent Equation (15).

**Figure 5:**
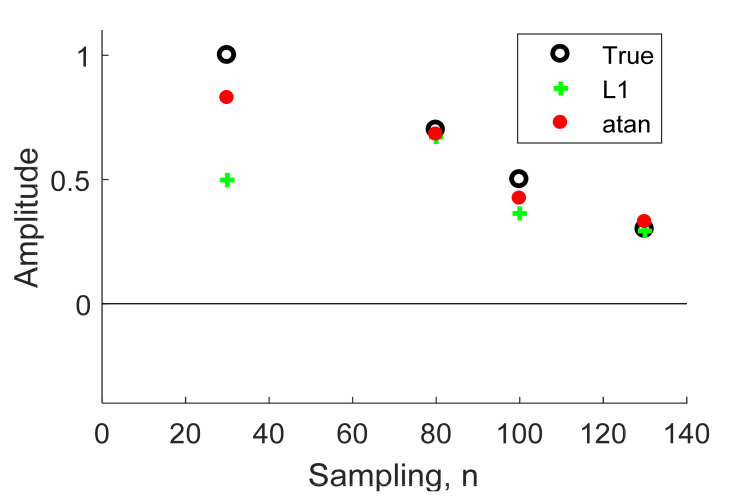
Comparison between the reflectivity series (True) and the estimated sparse signal using deconvolution with L1 norm and arctangent regularizations.

In Figure 4, the estimated sparse signal using the normal deconvolution with L2 norm regularization contained a great deal of noise, and was weak, rendering it inapplicable to real-world radar applications. Meanwhile, in Figure 5, we can see that the L1 norm solution is slightly attenuated compared with the arctangent solution, therefore, the sparse deconvolution using the arctangent regularization will be included as an efficient method to increase the detection performance for through-the-wall UWB radar, as described in the next section.

### 3.3. Through-Wall UWB Radar with Sparse Deconvolution Based on Arctangent

**Figure 6:**
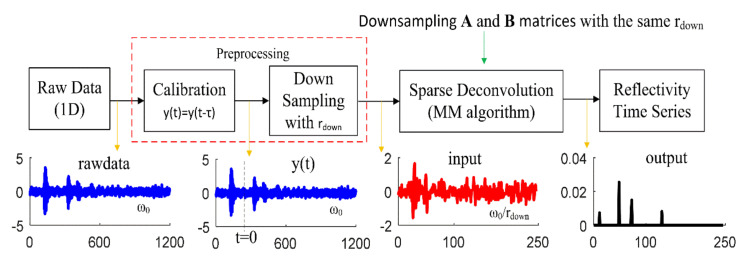
Block diagram of through-wall UWB radar with sparse deconvolution based on arctangent regularization.

The sparse deconvolution with arctangent regularization is so efficient in extracting the sparse signal from the noisy reflected signal (raw data) that lowpass, bandpass, and smooth filters are no longer required [16,17,18,19,20,21,22]. Figure 6 illustrates the steps for extracting the sparse signal for through-wall UWB radars.

**Figure 7:**
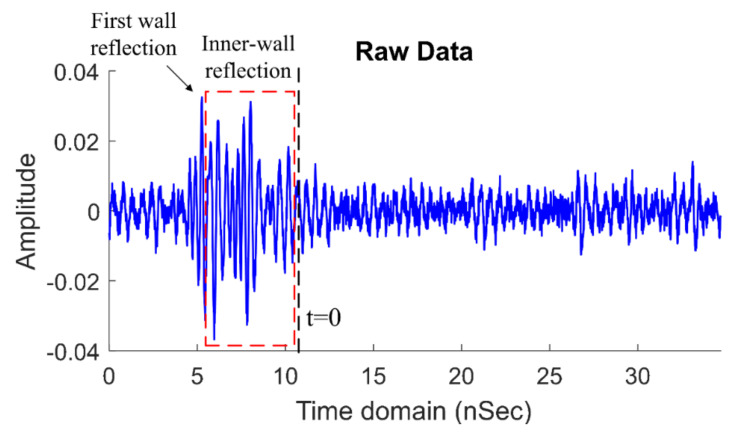
The pre-and post-calibration (beyond *t* = 0) received signal.

In the first step, the noisy received signal was calibrated to remove the unwanted signals: [42,43,44,45]. Figure 7 illustrates the pre- and post-calibration (beyond *t* = 0) received signal.

The calibrated received signal *y*(*t*) was mathematically expressed by

(28) $$y\left(t\right)=y\left(t-\tau \right)$$

where *τ* is the time index of unwanted signals (i.e., antenna coupling, upon-impact wall reflection, and inner-wall reflection), it can be calculated by

(29) $$\tau =t_{1}+3\;\times \;2\frac{D_{wall}}{v_{wall}},\;\;\mathrm{where}\;v_{wall}=\frac{1}{\sqrt{\mu_{0}\varepsilon_{0}\varepsilon_{r}}}$$

where *t*_1_ is the first wall reflection derived by “*findpeaks*” (Matlab command); factor 3 is a constant to allow sufficient time for upon-impact wall reflection and inner-wall reflection; factor 2 is round-trip delay; *D_wall_* is the wall thickness; *v_wall_* is the speed of the wave in the wall; *ε_r_* is the relative permittivity; and the permittivity (𝜀_0_) and permeability (𝜇_0_) of vacuum are 8.854187 × 10^−12^ F/m and 4𝜋 × 10^−7^ H/m [42]. Note that the singular value decomposition has been widely used to solve such problems, but it takes a long time to process during its matrix inversion.

Second, before using the sparse deconvolution, the calibrated signal was down sampled to avoid running out of memory throughout N × N matrix inversion (long calculations), given *r_down_* is the down sampling ratio under the Nyquist sampling condition. In this flow chart, the down sampling was done by the integer factor method (e.g., “downsample” in Matlab). Besides, the N × N band matrices (**A** and **B**) must also be down sampled by the same *r_down_*, and the normalized angular frequency (*ω*_0_) was recalculated by

(30) $$\omega_{0}=\frac{\omega_{c}}{f_{s}}=2\pi f_{c}\left(\Delta t*r_{down}\right)$$

where *f_c_* is the center frequency, ∆*t* is the time resolution of the analog-to-digital converter (ADC), and *r_down_* is the down sampling ratio.

## 4. Experiments with Human Subjects

**Figure 8:**
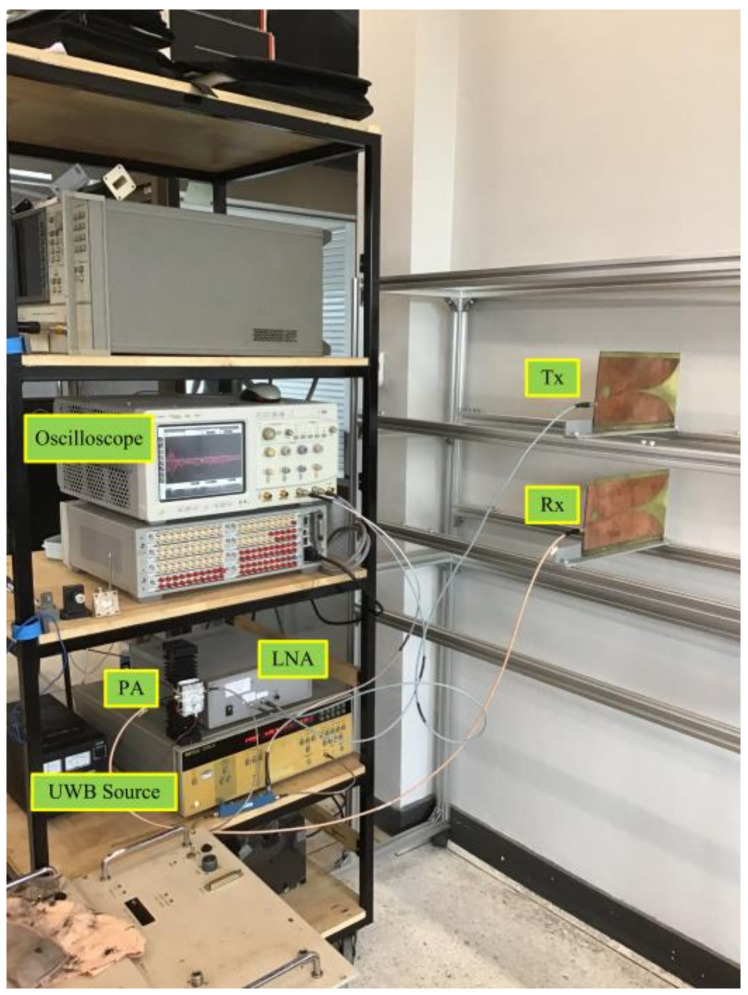
Experimental setup of the through-wall UWB radar.

Figure 8 depicts the experimental setup of the through-wall UWB radar scheme, corresponding to the block diagram in Figure 1. Table 1 tabulates the specifications of the experimental equipment and parameters.

The experiments were carried out with a concrete wall approximately 20 cm in thickness. The concrete wall was fashioned from three columns of concretes. In Figure 8, the S-band UWB pulse (2–4 GHz) from the UWB source was fed into the power amplifier (PA) and to the Vivaldi Tx antenna. The reflected signal received by the Vivaldi Rx antenna was amplified by the low-noise amplifier (LNA) and sent to the oscilloscope. The oscilloscope captured the received signal and transferred it to a computer via a GPIB port. The data were then discretized by MATLAB 2018a for sparse deconvolution.

### 4.1. Calibrating Recevied Signal

When the system obtains the received signal from the oscilloscope, as shown below (example at 3 m), it will be calibrated by using Equation (28) to remove the wall and antenna responses.

**Figure 9:**
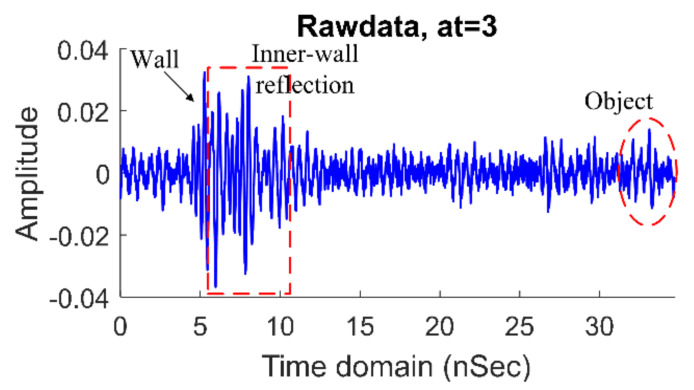
Rawdata at the human range of 3 m.

From Figure 9, the duration of the received signals captured by the oscilloscope was 35 ns, with a time resolution (∆*t*) of approximately 3.125 ps. The received data were discretized into 11,100 data points. According to the flow chart in Figure 6, the received signal was calibrated by Equation (28) with the zero-offset τ = 11 ns by computing Equation (29), with *t*_1_ = 6 ns (first wall reflection), *D_wall_* = 10 cm (the wall thickness), *ε_r_* = 4.5 (the relative permittivity) [47], and *v_wall_* = 1.4132 × 10^8^ m/s (the speed of the wave in the concrete wall).

This calibration is easy and provides quite accurate evaluation, but requires a lot of parameters to work, which is suitable for the known material and thickness of the wall. In a realistic context, with variable obstacles and, sometimes, no obstacle at all, the antenna and wall coupling problems can quickly be removed by observing the setting time shift of the received signal [48]. Note that the singular value decomposition has been widely used to solve such a problem, but it takes a long time to process during its matrix inversion.

Next, the calibrated signal was down-sampled by “downsample” command MATLAB, where the normalized frequency (*ω*_0_) was evaluated in Equation (30), *ω*_0_ = 2π3 × 10^9^ × 3.125 × 10^−12^ × 4 = 0.2356 rad/sample with *f_c_* = 3 GHz, ∆*t* = 3.125 ps, and *r_down_* = 4. The down-sampling directly reduced the calcaultion time of the inverse matrix of the sparse deconvolution, from (N × N) to (N/4 × N/4), dimension while maintaing the Nyquist sampling condition [37].

### 4.2. Sparse Deconvolution Results

Human range was extracted from the calibrated signal by using the sparse deconvolution based on the arctangent regularization, accoding to Algorithm 1. The **A** and **B** matrices in Algorithm 1 were calculated from Equations (3) and (4), fixed *r* = 0.9 and *ω*_0_ = 0.2356 rad/sample, respectively. To avoid nonconvex optimization, the deconvolution parameters λ and *α* were 0.4 and 0.9/λ, respectively, by evaluating the Equation (27) [37].

**Figure 10:**
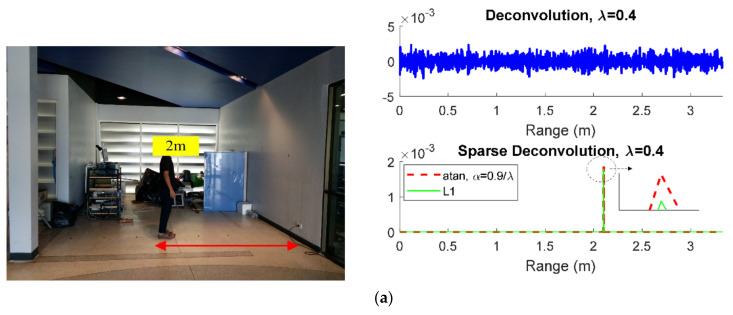

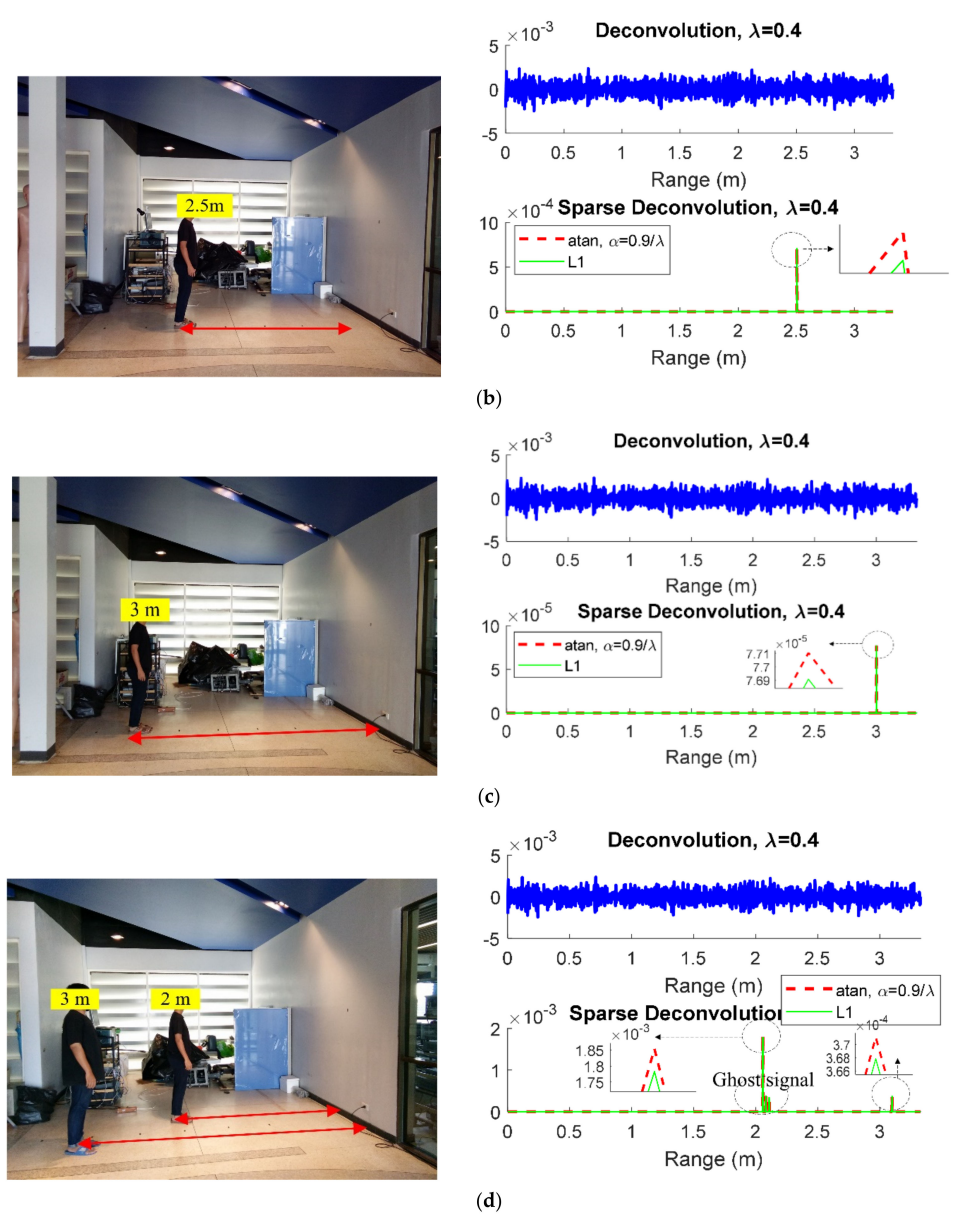

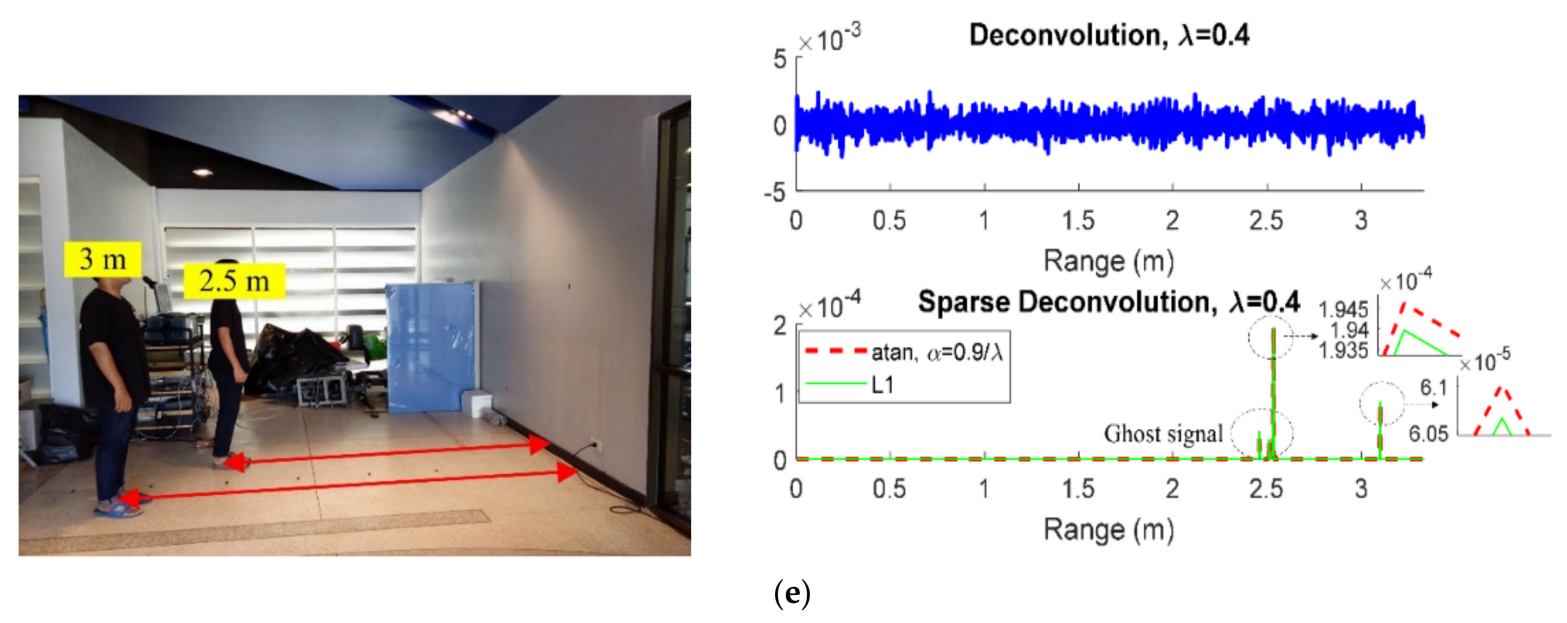
Sparse deconvolution based on arctangent regularization (red line) compared with L1 norm (green line) and L2 norm (blue line). (**a**) a distance of 2 m; (**b**) a distance of 2.5 m; (**c**) a distance of 3 m; (**d**) two persons at (2 m, 3 m); (**e**) two persons at (2.5 m, 3 m).

The experimental results, as shown in Figure 10, were compared with L1 and L2 regularizations by varying a person’s distance at 2, 2.5, and 3 m, as well as two person distances at (2 m, 3 m) and (2.5 m, 3 m).

In Figure 10, the human detection performance of the through-wall radar scheme with the ordinary deconvolution (L2 norm) was unsatisfactory, while that of the radar schemes with sparse deconvolution (L1 norm and arctangent regularizations) could effectively locate the human subjects behind the wall. By comparison, the arctangent regularization was significantly higher than that with L1 norm.

For the experiments with one participant standing at 2, 2.5, or 3 m (Figure 10a–c), the estimated behind-the-wall distances using sparse deconvolution with arctangent regularization were 2.1, 2.55, and 3.1 m, respectively. For the experiment with two participants standing (2 m, 3 m) and (2.5 m, 3 m) behind the wall (Figure 10d,e), the estimated behind-the-wall distances were (2.11 m, 3.2 m) and (2.55 m, 3.16 m). The distance error comes from the other effects of the wave propagation in the concrete wall. These issues are quite difficult to model, because the walls in real-world applications are inhomogeneous and not purely dielectric material [47,48].

Furthermore, for the experiment with two participants standing (Figure 10d,e), the ghost signal is probably difficult to remove due to the reflection of the electromagnetic wave scattering off the nearby participants. Technically, if both the wavelet **H** and the input signal **x** are unknown, this is the blind deconvolution problem. It estimates the wavelet model **H** of the subsurface layer and the transmitted pulse, which is useful for GPR and seismic data [4,5,23,24,25,26,27,28].

In previous works [43,44,48], UWB radar algorithms for human detection need to capture the received signals at least 512 times per minute (60 s), for over 1 cycle vital sign signal, with a Nyquist sampling condition. The sparse deconvolution algorithm is used for reconstruing human range from only one received signal, and for faster detection. However, with this method, it is difficult to distinguish between a standing human and static objects. In real-world applications, humans have motions, so the detectable range is sufficient for obtaining their positions [41]; sparse deconvolution could be deployed in various through-obstruction applications with faster detection, especially in hostage rescue operations.

## 5. Conclusions

Through-the-wall UWB radar posits that the unknown object time series **x** is sparse (range domain), and is solved by the sparse deconvolution based on the arctangent regularization to induce sparsity more strongly than the L1 norm. The cost function of this sparse deconvolution model is also composed of the band matrices **A** and **B**, which provide a fast solution by the majorization–minimization (MM) algorithm. Moreover, the S-band UWB radar is intended for locating human subjects behind a wall, with the following step-by-step description: (1) calibration; (2) down-sampling; (3) designing the band matrices. To validate this, the through-wall UWB radar scheme with deconvolution based on L2 norm, L1 norm, and arctangent regularizations was experimentally applied to detect human subjects at different behind-the-wall distances, and experimental results were compared. The results showed that the human detection performance of the radar scheme with L2 norm regularization was poor. On the other hand, the radar scheme with L1 norm and arctangent regularizations could effectively detect the human subjects behind a wall. Nevertheless, the human detection performance of the through-wall UWB radar scheme with arctangent regularization was significantly higher than that with L1 norm regularization.

## Figures and Tables

**Table 1:** Equipment List.

**Block**	**Manufacturer**	**Specifications**
UWB source	HP-8133A pulse generator	0.5 V Peak voltage, Central frequency 3 GHz
Tx and Rx antennas	Vivaldi type (S-band) [48]	2–5 GHz, 10 dBi, angular width (3 dB) ≈ 45
PA	ZVE-8G + Mini-Circuits	2–8 GHz, 30 dBm
LNA	R&K-AA260-OS	2–5 GHz, 26 dBm
ADC	Agilent Oscilloscope, Infiniium DSO80604B	Max frequency 6 GHz
USB port	Agilent GPIB, 82357B	Transfer over 850 KB/sec
Transmission power	-	−5 dBm, bandwidth of 2–5 GHz
Relative permittivity (*ε_r_*)	-	*ε_r_* = 4.5, concrete wall [47]
Thickness of the concrete wall	-	Approximately 10 cm

## Data Availability

Not applicable.
